# CliniMACS Prodigy Manufacturing of Switchable, AND-Gate CAR T Cells

**DOI:** 10.3390/ijms26115024

**Published:** 2025-05-23

**Authors:** Alexandra von Jutrzenka-Trzebiatowski, Rutuja Gupte, Cansu Daglar, Nicole Berndt, Claudia Arndt, Michael Bachmann, Anja Feldmann

**Affiliations:** 1 Helmholtz-Zentrum Dresden-Rossendorf (HZDR), Institute of Radiopharmaceutical Cancer Research, Bautzner Landstrasse 400, 01328 Dresden, Germany; a.von-jutrzenka-trzebiatowski@hzdr.de (A.v.J.-T.); r.gupte@hzdr.de (R.G.); c.daglar@hzdr.de (C.D.); n.berndt@hzdr.de (N.B.); c.arndt@hzdr.de (C.A.); 2 Mildred Scheel Early Career Center, Faculty of Medicine Carl Gustav Carus, TUD Dresden University of Technology, 01307 Dresden, Germany; 3 National Center for Tumor Diseases (NCT), NCT/UCC Dresden, a Partnership Between DKFZ, Faculty of Medicine and University Hospital Carl Gustav Carus, TUD Dresden University of Technology, and Helmholtz-Zentrum Dresden-Rossendorf (HZDR), 01307 Dresden, Germany; 4 German Cancer Consortium (DKTK), Partner Site Dresden and German Cancer Research Center (DKFZ), 69120 Heidelberg, Germany

**Keywords:** adapter CAR T cells, CliniMACS Prodigy, tumor therapy, RevCAR and Dual-RevCAR system

## Abstract

The Reverse Chimeric Antigen Receptor (RevCAR) system is an adapter CAR T cell technology that allows the precise tuning of T cell activity and, thus, improved safety management. RevCAR T cells recognize and eradicate tumor cells via a bispecific adapter molecule, termed the RevCAR Target Module (RevTM). To further reduce the risk of on-target off-tumor toxicities, Dual-RevCAR T cells can be employed. These cells harbor two different RevCAR constructs, with the signaling domain of either CD3zeta or CD28. Therefore, Dual-RevCAR T cells only exert their full function when both RevCAR constructs are triggered simultaneously upon recognition of two different tumor antigens via RevTMs, enabling a precise AND-gate targeting approach and rendering them highly interesting for clinical application. For this purpose, standardized and reproducible clinical-grade cell manufacturing is required, for which the CliniMACS Prodigy can be used. Here, we present that automated processing of RevCAR and Dual-RevCAR T cells via the CliniMACS Prodigy results in potent expansion, strong transduction, and a favorable phenotype for clinical application. Moreover, obtained cell products were highly functional in a strict RevTM-dependent manner for both monospecific and AND-gate targeting, clearly underlining their high potential for clinical application against various tumor entities.

## 1. Introduction

**Figure 1:**
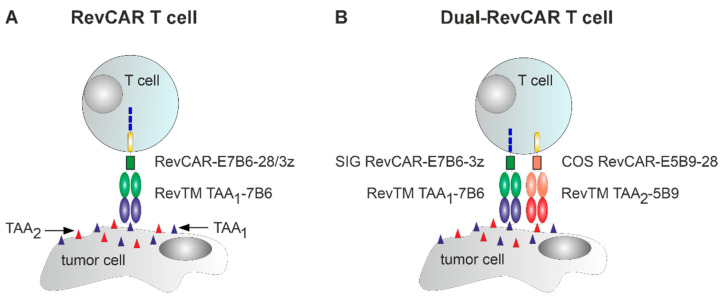
Schematic representation of the Reverse Chimeric Antigen Receptor (RevCAR) and Dual-RevCAR system. (**A**) Cross-linkage of a RevCAR T cell harboring a RevCAR-E7B6-28/3z to a tumor cell possessing two different tumor-associated antigens (TAAs) via a RevCAR Target Module (RevTM) with specificity for TAA_1_ and the E7B6 peptide epitope (RevTM TAA_1_-7B6) is schematically presented. Addition of or replacement with an alternative RevTM directed, for example, to TAA_2_ (RevTM TAA_2_-7B6) allows for either simultaneous or sequential targeting of two independent TAAs (OR-gate targeting). (**B**) Dual-RevCAR T cells contain two different RevCAR constructs: (i) a signaling (SIG) RevCAR with the CD3zeta signaling domain and the E7B6 peptide epitope as an extracellular binding domain (RevCAR-E7B6-3z) and (ii) a co-stimulatory (COS) RevCAR with the signaling domain of CD28 and the extracellular E5B9 peptide epitope (RevCAR-E5B9-28). For complete activation and functionality, both RevCAR constructs need to be simultaneously triggered via RevTM TAA_1_-7B6 and RevTM TAA_2_-5B9, enabling an AND-gate tumor targeting approach.

Since their first application in humans, Chimeric Antigen Receptor (CAR) T cells have revolutionized the field of cancer immunotherapy, showing remarkable efficiency, especially in hematological malignancies [1,2]. On the downside, however, CAR T cell treatment can still be accompanied by severe side effects, such as cytokine release syndrome and neurologic or on-target off-tumor toxicities [3]. To overcome these challenges, switchable adapter CAR technologies emerged, whereby the CAR does not directly recognize a tumor-associated antigen (TAA) but requires a specific adapter molecule for it. Thus, adapter CAR T cells can be turned “ON” and “OFF” depending on adapter molecule availability, which clearly increases their safety profile [4,5,6,7,8,9]. Adapter CAR technologies developed in our group are the Universal CAR (UniCAR) [5,10,11,12,13,14,15] and Reverse CAR (RevCAR) [16,17,18,19] approach, of which the UniCAR platform has already proven its safety and efficiency in patients with CD123-positive hematological malignancies (NCT04230265) [20,21]. Moreover, a clinical phase I trial investigating RevCAR T cells was recently initiated (NCT05949125). In contrast to classical (adapter) CAR T cells, RevCAR T cells lack an extracellular antibody (Ab)-based binding moiety but instead possess a peptide epitope (either E7B6 or E5B9 or both) [16] (Figure 1A and Supplementary Figure S1A). These peptide epitopes derived from the nuclear autoantigen La/SS-B are non-immunogenic even in autoimmune patients positive for anti-La/SS-B Abs [22,23]. For the cross-linkage with a target cell, RevCAR T cells require a bispecific adapter molecule, termed the RevCAR Target Module (RevTM). The RevTM consist of two single-chain fragment variables with specificity for a TAA and the respective RevCAR peptide epitope (Figure 1A) [16]. RevCAR T cells can not only be precisely controlled by RevTM availability but also directed against several TAAs by applying RevTMs with different TAA specificity (OR-gate targeting) [16,17,18], which reduces the risk of emerging tumor escape variants and helps to overcome tumor heterogeneity. However, keeping in mind that most TAAs are also expressed in normal tissue, although to a lesser extent [24,25], a targeting strategy sparing healthy cells needs to be further implemented. To address this requirement, we designed a Dual-RevCAR vector (Supplementary Figure S1B) containing two distinct RevCAR constructs: a signaling (SIG) RevCAR and a co-stimulatory (COS) RevCAR, with the extracellular peptide epitope E7B6 or E5B9 and the intracellular signaling domain of CD3zeta (SIG RevCAR-E7B6-3z) or CD28 (COS RevCAR-E5B9-28), respectively [16]. Importantly, T cells transduced with this Dual-RevCAR vector are only fully activated when both SIG RevCAR-E7B6-3z and COS RevCAR-E5B9-28 are stimulated simultaneously by their respective RevTMs, which therefore allows AND-gate targeting and increases the precision of a Dual-RevCAR T cell attack towards tumor cells (Figure 1B) [16,17,18,19]. So far, it was already demonstrated that RevCAR and Dual-RevCAR T cells potently kill tumor cells of various entities, including prostate cancer, acute myeloid leukemia, glioblastoma, and colorectal cancer [16,17,18,19]. Thus, altogether, RevCAR and Dual-RevCAR T cells are highly promising “living drugs” for the treatment of cancer. For application in patients, the manufacture of CAR T cells via the CliniMACS Prodigy is widely implemented [26,27,28,29,30]. This device allows for a GMP-compliant, automated, and standardized closed-system large-scale process and, therefore, reduces hands-on time, operator variation, and contamination risk [27,29]. Here, we present the manufacturing of RevCAR and Dual-RevCAR T cells via the CliniMACS Prodigy and provide extensive phenotypic and functional characterization of these cells.

## 2. Results

### 2.1. RevCAR and Dual-RevCAR T Cells Were Manufactured via the CliniMACS Prodigy with High Transduction Efficiencies and Expansion Rates

**Figure 2:**
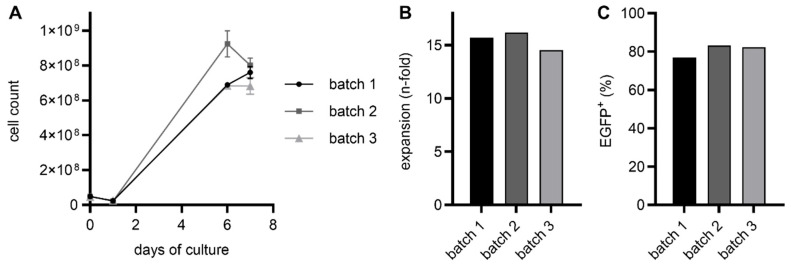
Cell growth and transduction rate of CliniMACS Prodigy-manufactured RevCAR T cells. By applying the CliniMACS Prodigy, T cells were isolated and activated on day 0 followed by transduction on day 1. Cells were allowed to expand until day 7. (**A**) Cell counts were measured at day 0, day 1, day 6, and day 7 after the harvest using flow cytometry, shown as the mean ± SD of at least duplicates. (**B**) Additionally, on day 7, cell expansion was assessed and (**C**) the transduction rate was determined using flow cytometry by measuring the signal of the marker gene EGFP, which strongly correlates with RevCAR surface expression.

**Figure 3:**
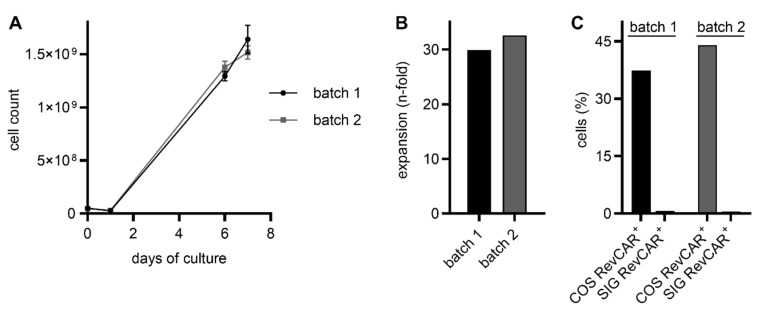
Cell growth and transduction rate of CliniMACS Prodigy-manufactured Dual-RevCAR T cells. T cells were isolated, activated (both at day 0), transduced (day 1), and cultured until day 7 using the CliniMACS Prodigy. (**A**) At day 0, day 1, day 6, and day 7 (after the harvest), the cell count was determined, shown as the mean ± SD of at least duplicates, (**B**) and the cell expansion until day 7 was calculated. (**C**) In addition, the CAR expression was assessed by staining with an anti-La monoclonal antibody (mAb) (5B9) (for COS RevCAR-E5B9-28) or anti-La mAb (7B6) (for SIG RevCAR-E7B6-3z) and a fluorescently labeled secondary goat anti-mouse IgG Ab and analyzed with flow cytometry at day 8.

Using the CliniMACS Prodigy, we successfully manufactured three RevCAR T cell products derived from different healthy T cell donors, which contain a RevCAR-E7B6-28/3z construct. After the harvest, total cell numbers ranging from 6.8 × 10^8^ to 8.0 × 10^8^ were achieved (Figure 2A), corresponding to an average expansion rate of 15.5-fold (14.5-fold to 16.2-fold, Figure 2B). In addition, genetic modification of T cells was highly efficient, reaching an average transduction rate of 80.7% (76.9% to 82.2%, Figure 2C). Besides RevCAR T cells only harboring the RevCAR-E7B6-28/3z construct, we also successfully generated Dual-RevCAR T cells via the CliniMACS Prodigy, which express both SIG RevCAR-E7B6-3z and COS RevCAR-E5B9-28. As shown in Figure 3A,B, both batches of Dual-RevCAR T cells potently expanded in the CliniMACS Prodigy, reaching an average cell number of 1.6 × 10^9^ (31.2-fold expansion). In line with previously published data of manually produced Dual-RevCAR T cells [16,17,18], SIG RevCAR-E7B6-3z and COS RevCAR-E5B9-28 were differentially expressed. COS RevCAR-E5B9-28 was present in about 40.7% of cells. In contrast, SIG RevCAR-E7B6-3z was barely detectable (0.6%, Figure 3C), which might be due to its very low density on T cells ranging from 15 [16] to 270 [18] receptors per cell.

### 2.2. CliniMACS Prodigy-Manufactured RevCAR and Dual-RevCAR T Cells Possess a Favorable Phenotype for Clinical Application

**Figure 4:**
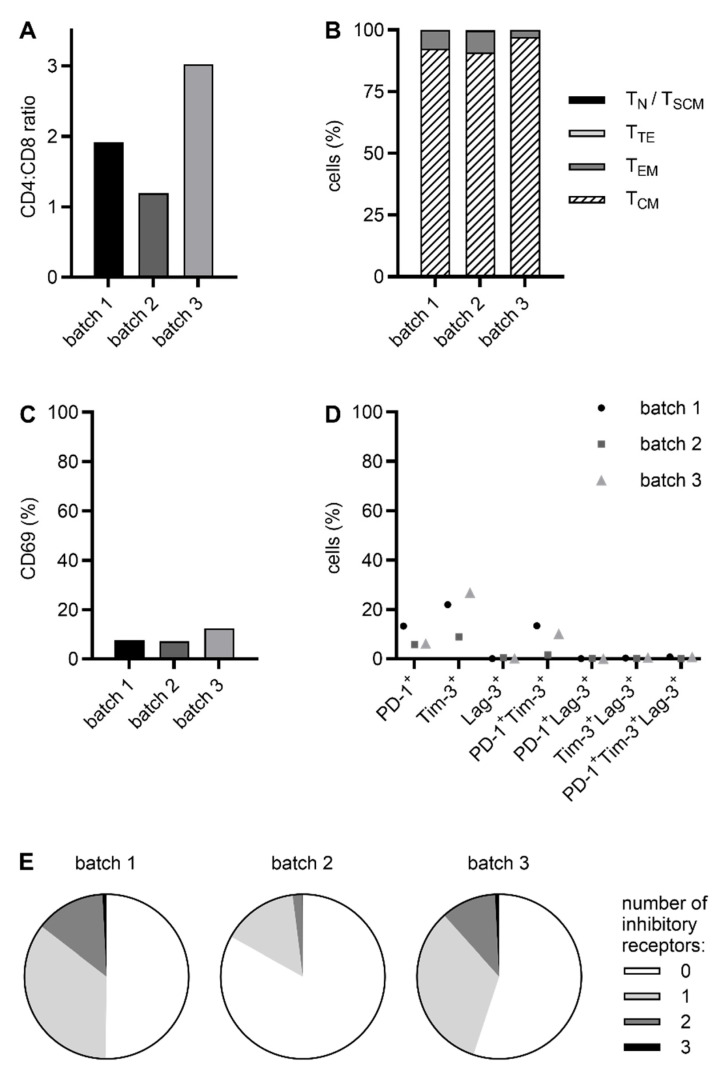
Phenotypic features of CliniMACS Prodigy-manufactured RevCAR T cells. RevCAR T cells were stained with mAbs against (**A**) CD4, CD8, (**B**) CD62L, CD45RO, (**C**) CD69, (**D**,**E**) PD-1, Tim-3, and Lag-3 for flow cytometric analysis. Data are shown for three individual donors. (**B**) T_N_/T_SCM_—naive/stem cell memory (CD62L^+^CD45RO^−^); T_CM_—central memory (CD62L^+^CD45RO^+^); T_EM_—effector memory (CD62L^−^CD45RO^+^); T_TE_—terminal effector (CD62L^−^CD45RO^−^).

**Figure 5:**
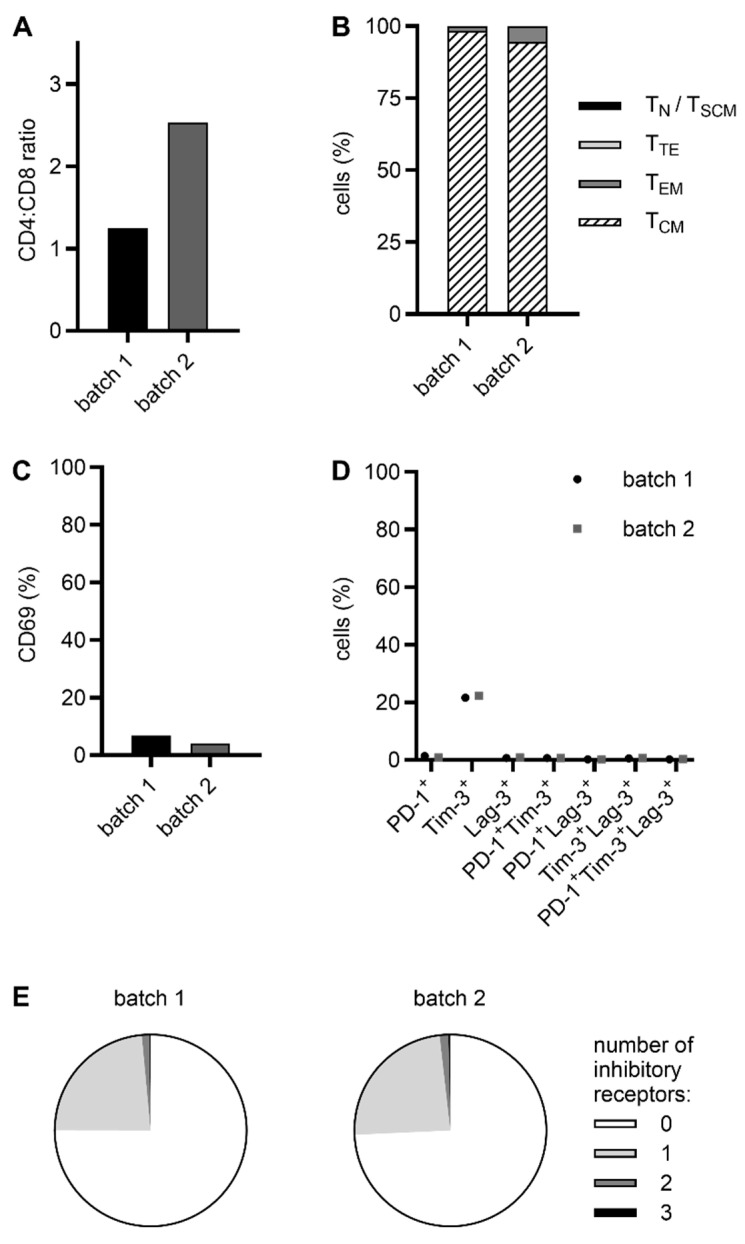
Phenotypic features of CliniMACS Prodigy-manufactured Dual-RevCAR T cells. Dual-RevCAR T cells were incubated with mAbs against (**A**) CD4, CD8, (**B**) CD62L, CD45RO, (**C**) CD69, (**D**,**E**) PD-1, Tim-3, and Lag-3 for 15 min at 4 °C in the dark and analyzed via flow cytometry. Data of two individual donors are presented. (**B**) T_N_/T_SCM_—naive/stem cell memory (CD62L^+^CD45RO^−^); T_CM_—central memory (CD62L^+^CD45RO^+^); T_EM_—effector memory (CD62L^−^CD45RO^+^); T_TE_—terminal effector (CD62L^−^CD45RO^−^).

To further characterize our RevCAR and Dual-RevCAR T cell products, we determined the CD4:CD8 ratio as well as the memory, activation, and exhaustion status. All five CAR T cell products exhibited a predominance of CD4 T cells over CD8 T cells (RevCAR T cells: average CD4:CD8 ratio of 2, Figure 4A; Dual-RevCAR T cells: average CD4:CD8 ratio of 1.9, Figure 5A). In addition, most of the cells showed a central memory T (T_CM_) cell phenotype (RevCAR T cells: 93.5%, Figure 4B; Dual-RevCAR T cells: 96.5%, Figure 5B), whereas only 6.4% of RevCAR T cells and 3.4% of Dual-RevCAR T cells were effector memory T (T_EM_) cells. Naive and stem cell memory T (T_N_/T_SCM_) cells and terminal effector T (T_TE_) cells were barely detectable. Moreover, only a minor percentage of cells expressed the activation marker CD69 (RevCAR T cells: 9.1%, Figure 4C; Dual-RevCAR T cells: 5.4%, Figure 5C) and the inhibitory receptors PD-1, Tim-3, and Lag-3 (RevCAR T cells: 8.4%, 19.2%, and 0.2%, respectively, Figure 4D; Dual-RevCAR T cells: 1.1%, 21.9%, and 0.7%, respectively, Figure 5D). Importantly, simultaneous expression of several inhibitory receptors, being a characteristic feature of exhausted cells [31,32], was marginal (RevCAR T cells: 8.8% and 0.6%, Figure 4D and Figure 4E; Dual-RevCAR T cells: 1.4% and 0.3%, Figure 5D and Figure 5E, for co-expression of two or three inhibitory receptors, respectively).

### 2.3. CliniMACS Prodigy-Manufactured RevCAR and Dual-RevCAR T Cells Potently Kill Tumor Cells and Efficiently Secrete Cytokines

**Figure 6:**
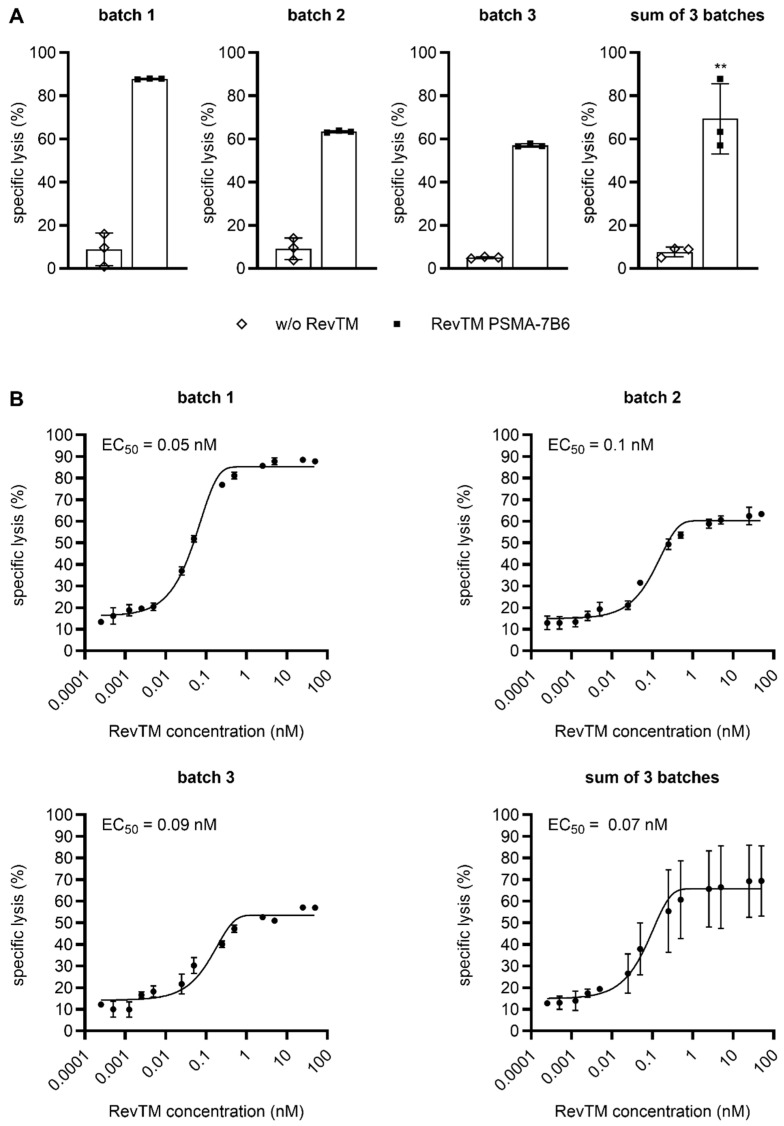
Highly potent and specific tumor cell lysis by CliniMACS Prodigy-manufactured RevCAR T cells. RevCAR T cells and PC3 cells expressing the prostate stem cell antigen (PSCA), prostate-specific membrane antigen (PSMA), and firefly luciferase (Luc) were incubated at an E:T ratio of 5:1 in the (**A**) absence (w/o RevTM) or presence of 50 nM or (**B**) increasing RevTM PSMA-7B6 concentrations. After 7 h, specific lysis was assessed. For this purpose, 50 µL per well of One-Glo™ Luciferase Reagent was added, and the luminescent signal was acquired using a luminometer. Sum of triplicates or three individual donors are shown (mean ± SD). Statistical analysis was performed on summarized data of the three individual donors (sum of three batches) using an unpaired, two-tailed Student’s *t*-test (** *p* ≤ 0.01 with respect to w/o RevTM).

**Figure 7:**
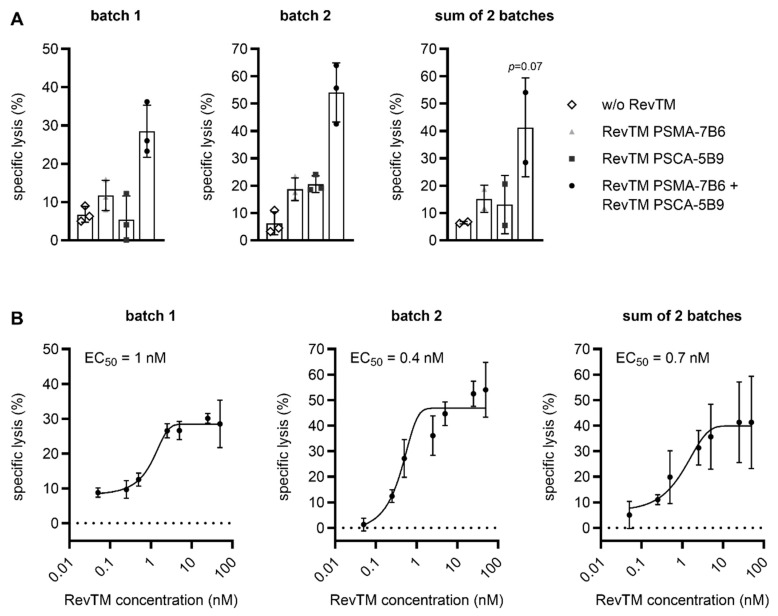
Highly potent and specific tumor cell lysis by CliniMACS Prodigy-manufactured Dual-RevCAR T cells. PC3 PSCA PSMA Luc cells were cultured with Dual-RevCAR T cells at an E:T ratio of 5:1 either (**A**) alone (w/o RevTM), together with 50 nM of the indicated RevTM(s), or (**B**) with an increasing concentration of RevTM PSMA-7B6 and RevTM PSCA-5B9 added simultaneously. By using a luminescence-based assay, specific lysis was measured after 15 h. Summarized data of triplicates or two individual donors are depicted (mean ± SD). Statistical analysis was carried out on summarized data of the two individual donors (sum of two batches) by applying a one-way ANOVA with Dunnett’s multiple comparisons test (*p* = 0.07 with respect to w/o RevTM).

Finally, all five CliniMACS Prodigy-produced RevCAR and Dual-RevCAR T cell products were functionally evaluated by assessing their cytotoxic potential (Figure 6 and Figure 7) and ability for cytokine secretion (Supplementary Figures S2 and S3). For this purpose, a well-established approach targeting the prostate stem cell antigen (PSCA) and prostate-specific membrane antigen (PSMA) [16] was applied exemplarily. T cells were cultured together with prostate cancer cells expressing the PSCA, PSMA, and firefly luciferase (Luc), either in the absence or presence of the indicated RevTM(s). As shown in Figure 6A, RevCAR T cells expressing the RevCAR-E7B6-28/3z construct efficiently eliminated tumor cells, which was strictly dependent on the availability of a cross-linking RevTM PSMA-7B6. In the case of Dual-RevCAR T cells, potent tumor cell killing only occurred in the simultaneous presence of the RevTM PSMA-7B6 and the RevTM PSCA-5B9 stimulating SIG RevCAR-E7B6-3z and COS RevCAR-E5B9-28, respectively (Figure 7A). In contrast, in comparison to the control without the RevTM, Dual-RevCAR T cells showed no or only negligible tumor cell eradication when either RevTM PSMA-7B6 or RevTM PSCA-5B9 was added alone to co-cultures. These data, on the one hand, prove that both SIG RevCAR-E7B6-3z and COS RevCAR-E5B9-28 are present on Dual-RevCAR T cells and, on the other hand, clearly demonstrate that AND-gate targeting by both RevTMs is required for Dual-RevCAR T cells to potently exert their cytotoxic activity. By titrating either RevTM PSMA-7B6 in the presence of RevCAR T cells (Figure 6B) or both RevTM PSMA-7B6 and RevTM PSCA-5B9 in the case of Dual-RevCAR T cells (Figure 7B), a half-maximal effective concentration (EC_50_) value of 70 pM and 700 pM was determined, respectively. Thus, CliniMACS Prodigy-manufactured RevCAR and Dual-RevCAR T cells are highly functional and kill even at picomolar RevTM concentrations.

In line with these results, secretion of TNF, IL-2, and IFN-γ only occurred upon cross-linkage of PSCA^+^PSMA^+^ tumor cells to either RevCAR T cells via the RevTM PSMA-7B6 (Supplementary Figure S2A) or Dual-RevCAR T cells via both RevTM PSMA-7B6 and RevTM PSCA-5B9 (Supplementary Figure S3A). In addition, cytokine release was triggered in a RevTM dose-dependent manner and even at picomolar or low-nanomolar RevTM concentrations (RevCAR T cells: TNF: EC_50_ = 0.7 nM, IL-2: EC_50_ = 0.9 nM, IFN-γ: EC_50_ = 0.6 nM, Supplementary Figure S2B; Dual-RevCAR T cells: TNF: EC_50_ = 2.0 nM, IL-2: EC_50_ = 1.8 nM, IFN-γ: EC_50_ = 2.0 nM; Supplementary Figure S3B).

Taken together, CliniMACS Prodigy-manufactured RevCAR and Dual-RevCAR T cells not only show a favorable phenotype for a clinical application but also possess high specificity and efficiency for tumor cell destruction and cytokine secretion.

## 3. Discussion

RevCAR and Dual-RevCAR T cells are highly promising platform technologies against cancer due to their efficient tumor cell killing and controllability by TAA-specific RevTMs [16,17,18,19]. Thus, they allow for better safety management and targeting of tumor escape variants by adding RevTMs with different TAA specificity (OR-gate targeting). Moreover, by applying Dual-RevCAR T cells, an even more precise attack and less on-target off-tumor toxicities can be achieved, as they need two TAAs simultaneously to fully exert their cytotoxic activity (AND-gate targeting) [16,17,18,19]. In the present study, we confirm that these RevCAR and Dual-RevCAR T cells can be successfully manufactured using the CliniMACS Prodigy. For this procedure, a protocol lasting 8 days was applied, allowing a fast supply of CAR-modified T cells to patients. Despite the short ex vivo cultivation time, a strong expansion of T cells was gained (Figure 2B and Figure 3B), comparable to the data of other groups also using an 8- or 9-day manufacturing protocol [27,28,30]. Moreover, transduction efficiency was high (Figure 2C and Figure 3C), especially in the case of RevCAR T cells reaching a percentage of genetically altered cells of about 80%, which is remarkable in comparison to transduction rates (19–66%) usually obtained by applying the CliniMACS Prodigy [26,27,28,29,30] and might be due to the use of spinoculation. Although Dual-RevCAR T cells were transduced to a lesser extent, they expanded to a greater extent than RevCAR T cells. Thus, in all five products, the number of genetically modified T cells exceeded 500 million, which will be sufficient for clinical application in patients. Since the phenotypic composition of a CAR T cell product has an impact on treatment outcome [33,34,35,36,37,38], we analyzed the CD4:CD8 ratio, memory status, activation, and exhaustion profile of the cells. As also reported in other studies [26,27,28], we detected a predominance of CD4 T cells over CD8 T cells (Figure 4A and Figure 5A). This observation might be attributable to the short ex vivo expansion [28], addition of human serum to the culture media [39], and/or supplementation of IL-7 and IL-15, as presumed by others [27,28]. Moreover, all five CliniMACS Prodigy-produced RevCAR and Dual-RevCAR T cell products mainly contained cells with a T_CM_ phenotype (Figure 4B and Figure 5B). For these cells, superior antitumor activity [40] and persistence, including migration to memory T cell niches, [41] was reported in mice and macaques, respectively. Furthermore, in cancer patients receiving an infusion product with an enriched T_CM_ count, a longer persistence [35,36,37] and higher expansion [38] of CAR T cells was observed, correlating with better treatment outcomes [35,36,37,38]. Besides the memory status, the exhaustion of CAR T cells also has an impact on therapy response. Fraietta et al. reported that cells simultaneously expressing PD-1 and Tim-3 or Lag-3 were enriched in infusion products of partially and non-responding patients with chronic lymphocytic leukemia in comparison to patients achieving complete remission [34]. In another clinical trial, co-expression of Tim-3 and Lag-3 of CAR T cells in the infusion product correlated with failure to reach an early molecular response in patients with large B cell lymphoma [33]. In light of these findings, it is of great relevance that the RevCAR and Dual-RevCAR T cell products investigated in our study possess not only a high abundance of T_CM_ cells but also a negligible expression of multiple inhibitory receptors in one cell (Figure 4D,E and Figure 5D,E). Consequently, their phenotype should be favorable for clinical application. Besides their phenotypic characteristics, the functionality and specificity of all five CliniMACS Prodigy-manufactured RevCAR and Dual-RevCAR T cell products were confirmed by their demonstrated strong cytotoxic activity (Figure 6 and Figure 7) and cytokine secretion (Supplementary Figures S2 and S3) only upon cross-linkage with tumor cells via RevTM PSMA-7B6 either alone (RevCAR T cells) or in combination with RevTM PSCA-5B9 (Dual-RevCAR T cells). The latter case represents an AND-gate targeting approach that further increases the specificity of a CAR T cell attack and reduces the risk for on-target off-tumor-toxicities, since two TAAs need to be present simultaneously. In this context, we believe that low expression of SIG RevCAR-E7B6-3z (Figure 3C), which might be due to the RevCAR structure itself, clearly favors the AND-gate targeting approach, as the CD3zeta signal alone seems to be insufficient for a potent T cell activation. In previous studies, we have shown that RevTMs of different sizes and, therefore, pharmacological properties can be combined [18,19], which will allow for an optimal tuning of Dual-RevCAR T cell performance depending on patient characteristics and disease stage. Thus, CliniMACS Prodigy-manufactured RevCAR and Dual-RevCAR T cells are highly promising “living drugs” for various tumor entities.

## 4. Material and Methods

### 4.1. Cell Lines

PC3 and 3T3 cells were obtained from the American Type Culture Collection and previously genetically modified to express the PSCA, PSMA, and Luc (PC3 PSCA PSMA Luc cells) [42] or the respective RevTMs (3T3 RevTM PSMA-7B6 cells; 3T3 RevTM PSCA-5B9 cells) [16]. Culture was performed in RPMI complete media for PC3 cells and in DMEM complete media for 3T3 cells [14]. All cells were kept in a humidified atmosphere at 37 °C and 5% CO_2_.

### 4.2. TM Expression and Purification

For RevTM production, 3T3 cells were seeded at a density of 2 × 10^6^ in 50 mL. After 96 h, the supernatant was collected. RevTMs were isolated via their His-tag and dialyzed overnight, with RevTM concentration and purity determined using SDS-PAGE, all as previously described in detail [12].

### 4.3. PBMC Isolation

Buffy coats were obtained from the German Red Cross Blood Donation Service North-East (Dresden, Germany) from healthy volunteers after giving informed consent. PBMCs were isolated either with Pancoll gradient centrifugation, seeded at a density of 2 × 10^6^/mL in RPMI complete media supplemented with 50 U/mL IL-2 (Human IL-2 IS, premium grade, Miltenyi Biotec GmbH, Bergisch Gladbach, Germany) and cultured over night, or using the CliniMACS Prodigy (Miltenyi Biotec GmbH).

### 4.4. Manufacturing of RevCAR and Dual-RevCAR T Cells Using the CliniMACS Prodigy

RevCAR and Dual-RevCAR T cells were manufactured via the CliniMACS Prodigy, using the tubing set TS520 (Miltenyi Biotec GmbH) and an automated process modified from the T cell transduction protocol. For this purpose, either buffy coats or PBMCs were loaded on the CliniMACS Prodigy, and isolation of CD4^+^ and CD8^+^ cells was performed using CliniMACS CD4 reagent, CliniMACS CD8 reagent, and CliniMACS PBS/EDTA buffer (all obtained from Miltenyi Biotec GmbH) containing 0.5% human serum albumin (PAN-Biotech GmbH, Aidenbach, Germany). At day 0, 50 × 10^6^ gained cells were used as a starting population, cultured in TexMACS GMP complete media (TexMACS GMP media supplemented with 12.5 ng/mL MACS GMP recombinant human IL-7, 12.5 ng/mL MACS GMP recombinant human IL-15 (all purchased from Miltenyi Biotec GmbH), and 3% human serum (HS, PAN-Biotech GmbH)) and activated using MACS GMP T cell TransAct (Miltenyi Biotec GmbH). The next day, lentiviral particles produced as previously described [43,44] were added to the culture followed by a spinoculation of 2 h. At day 3, cells were washed and the shaker (type 2) was activated. On day 5, 50 mL TexMACS GMP complete media was added. On day 7, final cell products were harvested in TexMACS GMP media containing 3% HS. Cells were either directly cryopreserved or cultured in RPMI complete media overnight and, subsequently, used for phenotypic and functional analysis.

### 4.5. Flow Cytometry

For phenotypic analysis, 0.2 × 10^6^ cells were stained with monoclonal Abs (mAbs) against CD4, CD8, CD45RO, CD69, PD-1, Tim-3, Lag-3 (all purchased from Miltenyi Biotec GmbH), and CD62L (BioLegend, San Diego, CA, USA) for 15 min. To determine the percentage of cells expressing the respective RevCAR construct, 0.2 × 10^6^ cells were incubated with 0.25 µg of the anti-La mAb (5B9) [45] or the anti-La mAb (7B6) [46] for 1 h, followed by staining with a fluorescently labeled secondary goat anti-mouse IgG Ab (BioLegend) for 30 min. All staining steps were performed at 4 °C in the dark. Stained cells were analyzed by applying MACSQuant^®^ Analyzer 10 and MACSQuantify^®^ software (both from Miltenyi Biotec GmbH).

### 4.6. Luminescence-Based Cytotoxicity Assay

To determine the cytotoxic potential of Prodigy-produced RevCAR and Dual-RevCAR T cells, luminescence-based cytotoxicity assays were performed as previously outlined in detail [15]. In brief, PC3 PSCA PSMA Luc cells were either cultured alone (Min), together with 5% Triton-X100 (Max), or with T cells at an effector-to-target cell (E:T) ratio of 5:1 (sample). In the case of RevCAR T cells, these co-cultures were either conducted without a RevTM, with 50 nM of RevTM PSMA-7B6, or with increasing concentrations of RevTM PSMA-7B6. In co-cultures with Dual-RevCAR T cells, 50 nM or increasing concentrations of RevTM PSMA-7B6 and RevTM PSCA-5B9 were added simultaneously. In addition, co-cultures without RevTMs and co-cultures only containing 50 nM of either RevTM PSMA-7B6 or RevTM PSCA-5B9 were used as controls. After 7 h or 15 h, respectively, cell-free supernatants were harvested for analysis via an ELISA (see Section 4.7). After 50 µL One-Glo™ Luciferase Reagent (Promega GmbH, Walldorf, Germany) was added to each well, data were acquired using a luminometer, and specific lysis was calculated according to the following equation: specific lysis (%) = ((Min − sample)/(Min − Max)) * 100. Using GraphPad Prism 10 software (GraphPad Software Inc., La Jolla, CA, USA), dose–response curves and EC_50_ values were determined.

### 4.7. Enzyme-Linked Immunosorbent Assay

The amount of IL-2, IFN-γ, and TNF secreted by RevCAR or Dual-RevCAR T cells was assessed using a sandwich ELISA. For this purpose, cell-free supernatants (see Section 4.6) were processed using a BD OptEIA™ Reagent Set B and a BD OptEIA™ Human IL-2 or IFN-γ or TNF ELISA Set (all purchased from BD Biosciences, Heidelberg, Germany) as outlined in the manufacturer’s instructions. In addition, a standard curve (range from 500 pg/mL to 7.8 pg/mL) was included. Data were acquired using a GloMAX^®^ Explorer System GM3500 (Promega GmbH). Values underneath the detection limit were considered to be 0. Dose–response curves and EC_50_ values were assessed by applying GraphPad Prism 10 software (GraphPad Software Inc.).

### 4.8. Statistical Analysis

Statistical analysis was performed via GraphPad Prism 10 software (GraphPad Software Inc.). When comparing two groups an unpaired, two-tailed Student’s *t*-test was applied, whereas a one-way ANOVA with Dunnett’s multiple comparisons test (with respect to the control “w/o RevTM”) was used in case of three or more groups. The respective statistical test is mentioned in the figure legends. *p*-values lower than 0.05 were determined as statistically significant.

## Data Availability

The original contributions presented in this study are included in the article/Supplementary Material. Further inquiries can be directed at the corresponding author(s).

## Supplementary Materials

The following supporting information can be downloaded at: https://www.mdpi.com/article/10.3390/ijms26115024/s1.
