# Supplementary material for: CliniMACS Prodigy Manufacturing of Switchable, AND-Gate CAR T Cells
Source: Int J Mol Sci. 2025 May 23;26(11):5024. doi: 10.3390/ijms26115024 (PMC12154027; doi:10.3390/ijms26115024)
Supplement: Supplementary file 1 [file ijms-26-05024-s001.zip › ijms-3647670-supplementary.pdf]

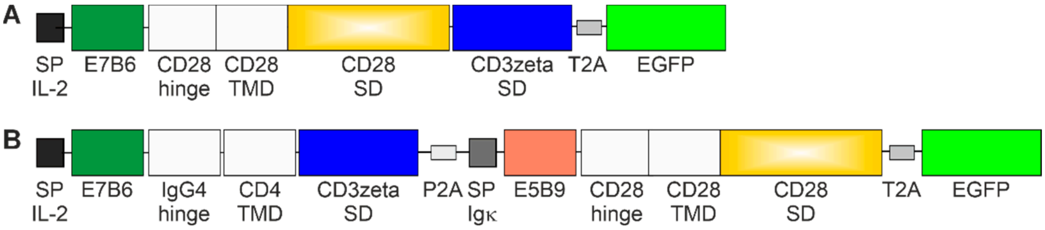

Supplementary Figure S1: Schematic representation of the RevCAR and Dual-RevCAR construct. (**A**) The RevCAR construct contains a signal peptide (SP) of human IL-2, the peptide epitope E7B6, the hinge, the transmembrane domain (TMD) and signaling domain (SD) of CD28, the SD of CD3zeta, a *Thosea asigna* virus 2A (T2A) site, and the marker gene EGFP. (**B**) The Dual-RevCAR construct is composed of a SP of human-IL-2, the peptide epitope E7B6, an IgG4 hinge, a CD4 TMD, and the SD of CD3zeta followed by a *porcine teschovirus*-1 2A (P2A) site, an Igκ SP, the peptide epitope E5B9, the hinge, the TMD and SD of CD28 as well as a T2A site, and EGFP.

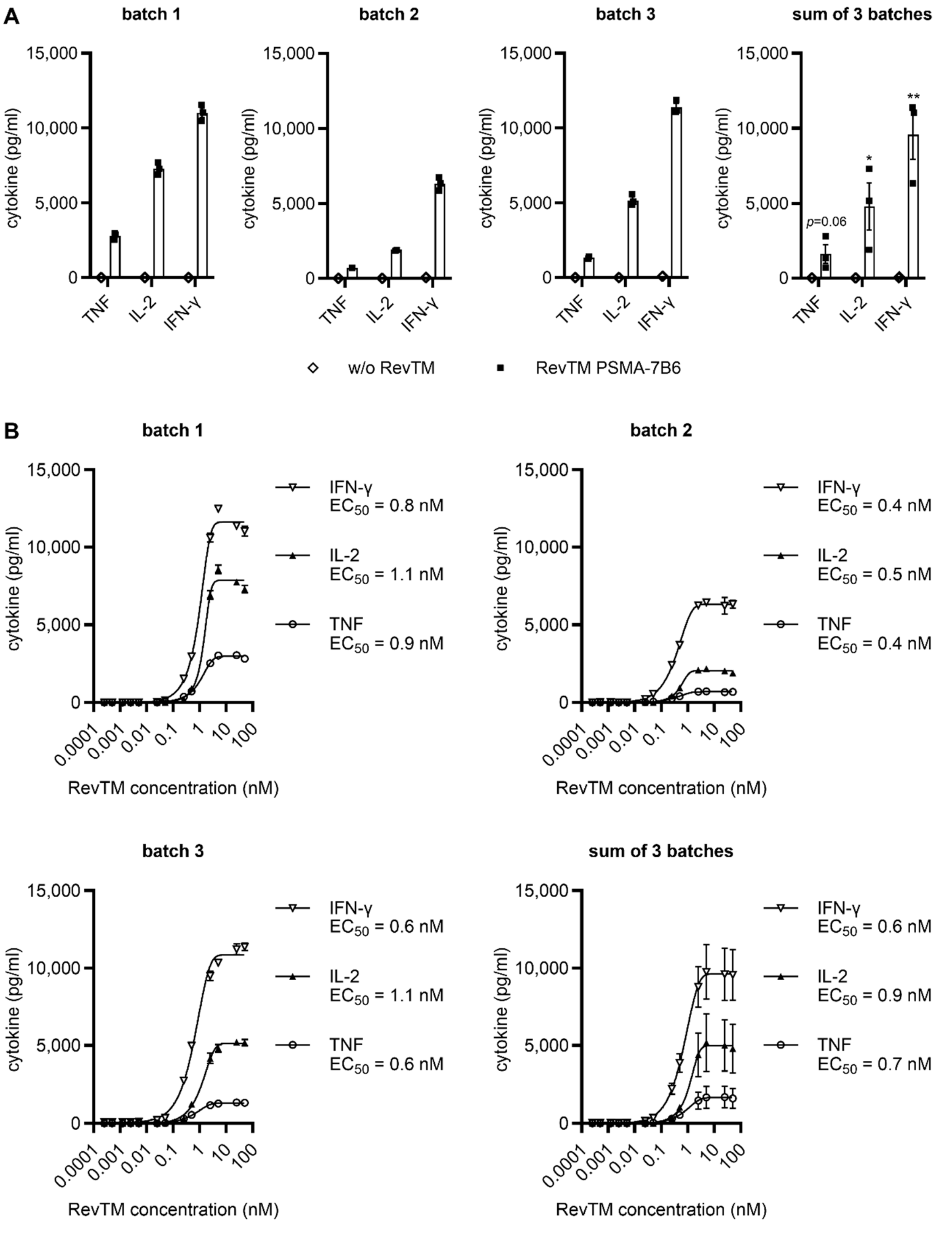

Supplementary Figure S2: Cytokine secretion of CliniMACS Prodigy-manufactured RevCAR T cells upon RevTM PSMA-7B6-mediated cross-linkage with tumor cells. RevCAR T cells and PC3 PSCA PSMA Luc cells were co-cultured (E:T of 5:1) (**A**) without RevTM addition (w/o RevTM) or in the presence of either 50 nM or (**B**) increasing RevTM PSMA-7B6 concentrations. After 7 h, supernatants were harvested and analyzed via an ELISA to determine the concentration of TNF, IL-2, and IFN-γ. Summarized data of triplicates or three individual donors are depicted as mean ± SEM. Statistical results were achieved by applying an unpaired, two-tailed Student´s *t*-test on summarized data of the three individual donors (sum of 3 batches) (** $p \leq 0.01$, * $p \leq 0.05$, $p = 0.06$ with respect to w/o RevTM).

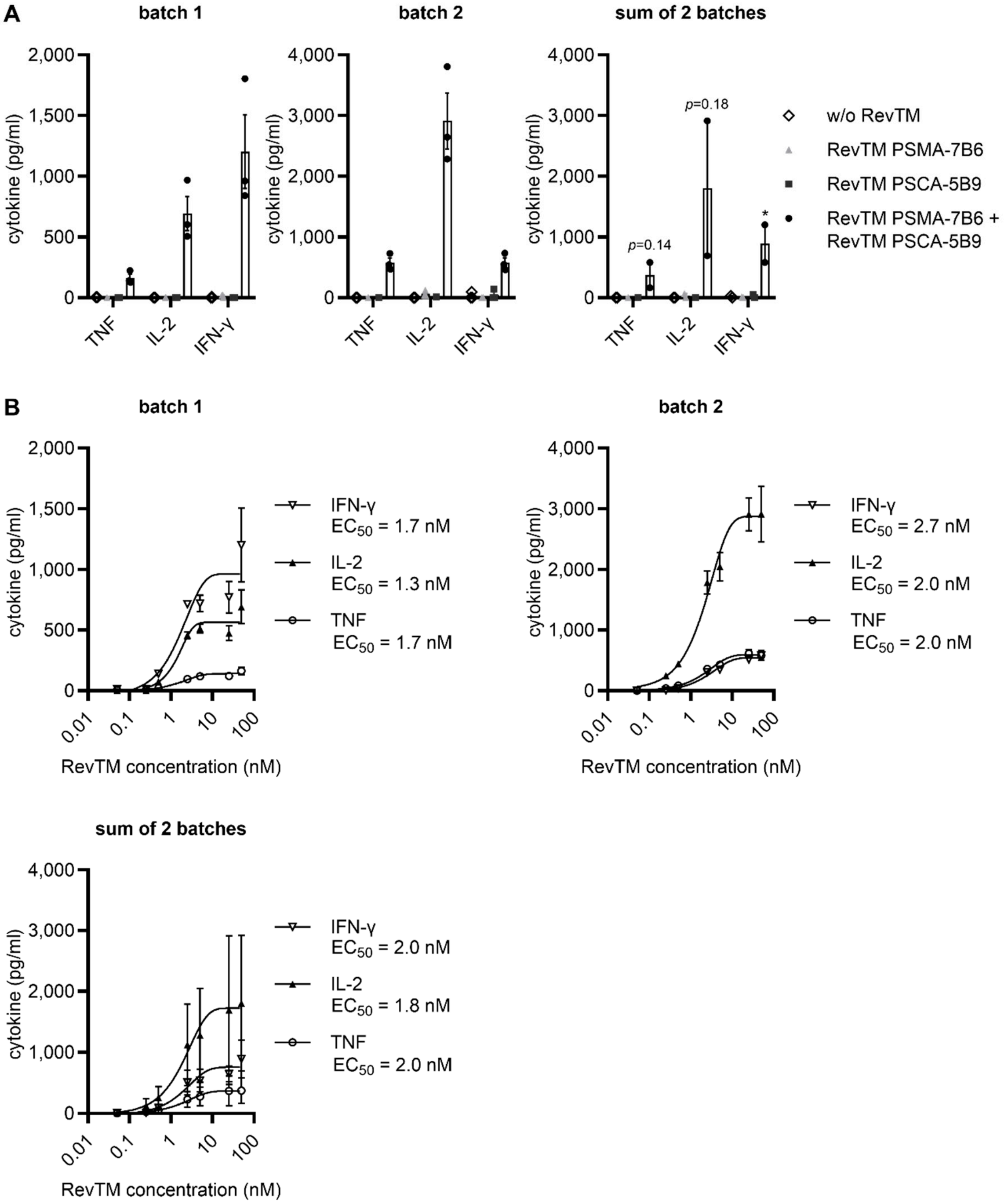

Supplementary Figure S3: Cytokine secretion of CliniMACS Prodigy-manufactured Dual-RevCAR T cells upon RevTM PSMA-7B6 and RevTM PSCA-5B9-mediated cross-linkage with tumor cells. Co-cultures of PC3 PSCA PSMA Luc cells and CliniMACS Prodigy-manufactured Dual-RevCAR T cells (E:T of 5:1) were performed either (**A**) in the absence (w/o RevTM) or presence of 50 nM of indicated RevTM(s) (**B**) or increasing concentrations of RevTM PSMA-7B6 and RevTM PSCA-5B9 for 15 h. Subsequently, supernatants were analyzed for secretion of TNF, IL-2, and IFN-γ using an ELISA. Summarized data of triplicates or two individual donors are shown as mean ± SEM. Statistical analysis was performed on summarized data of the two individual donors (sum of 2 batches) applying a one-way ANOVA with Dunnett's multiple comparisons test (* $p \leq 0.05$, $p = 0.14$, $p = 0.18$ with respect to w/o RevTM).
